# Protocol for a mixed methods longitudinal enquiry into the impact of a community based supportive service for people affected by cancer

**DOI:** 10.1186/s12885-016-2757-4

**Published:** 2016-09-06

**Authors:** Austyn Snowden, Jenny Young, Mick Fleming

**Affiliations:** Edinburgh Napier University, Sighthill Campus, Edinburgh, EH11 4BN Scotland, UK

**Keywords:** Cancer, Holistic needs assessment, Community care, Mixed methods, Social support, Quality of life, Patient activation, Health status, Link officer

## Abstract

**Background:**

Globally, cancer rates are increasing. In Scotland, it is estimated that 2 in 5 people will develop cancer in their lifetime. Therefore, this is crucial time to provide personalised care and support to individuals affected by cancer. In response to this a community based supportive cancer service was launched in Glasgow, Scotland. The aim of this service is to proactively provide those affected by cancer with an assessment of their needs and personalised support where needed. To our knowledge, there is no other service like this in the United Kingdom.

**Methods:**

The aim of this study is to understand if and how the service impacts upon the experiences and outcomes of people living with and affected by cancer. The study uses a sequential mixed methods design across a 5 year time point. Data gathering includes questionnaires, interviews, observations and reflective diaries. Participants include people affected by cancer who have used the service, a comparative sample who have not used the service, individuals who deliver the service and wider stakeholders. Outcomes include measures of patient activation, quality of life, health status, and social support. Data collection occurs at baseline, 2.5 years and 4 years with data from observations and reflective diaries supplemented throughout.

**Discussion:**

This study evaluates an innovative community based cancer service. It focuses on impact and process issues relevant to a) the individuals in receipt of the service, b) the service providers, and c) the wider culture. As the programme evolves overtime, the research has been designed to draw out learning from the programme in order to support future commissioning both within Scotland and across the UK.

## Background

Globally cancer is a leading cause of death. The World Health Organisation (WHO) estimated there were 14.1 million new cases of cancer in 2012, predicting this figure will rise by a further 20 % to 22 million within the next 20 years [1]. International and national figures show specific and consistent trends in terms of the increasing incidence, prevalence, and survival rates for people with cancer [2]. An ageing population, socio-economic factors and the adoption of lifestyle behaviours such as smoking, drinking alcohol, poor diet and physical inactivity all contribute to the increase of cancer cases [3].

In the United Kingdom (UK), cancer incidence rates have increased by more than a quarter over the last 40 years [2]. Prevalence of different risk factors varies by region and country. In particular, in Scotland cancer survival rates are low in comparison to the rest of Western Europe. This has been partly attributed to the late presentation of cancer and the high rates of lung cancer [4, 5]. Every year, approximately 30,000 people are told they have cancer in Scotland [6]. The predicted increase in the incidence of new cases of cancer in Scotland will be 33 % over the next 15 years resulting in over 40, 000 new cases per year between by the years 2023–2027 [7].

Risk factors relating to socio-economic factors and smoking have particular relevance for the Scottish city Glasgow. Glasgow is the biggest city in Scotland and the third biggest in the UK. The link between socio-economic factors, poor health and incidences of cancer are pertinent for the city. Deprivation figures show that Glasgow has 49 % of its total neighbourhoods categorised within the first and second most deprived quintiles [8]. Mortality rates have been found to be significantly higher (30 %) than the rate for other equally deprived cities in the UK such as Liverpool and Manchester [9]. With these factors in mind it was a priority to situate a new cancer initiative within the communities of Glasgow.

Individuals affected by cancer commonly experience some form of burden and distress [10]. Furthermore, a diagnosis of cancer not only impacts the person who is diagnosed but those close to them too [11]. Recognition of the need to screen for distress and provide personalised support services has grown and is a feature of current international policy and clinical practice guidelines [12–14]. Health policy within both the UK and Scotland has moved its emphasis from disease based only models to a person centred approach [15, 16]. Policy recommendations centre on supporting individual needs with the aim of acknowledging not only physical but also social and emotional needs. A number of key values; empowerment, inclusion, joint decision making and holistic needs assessment are enshrined within the definition of personalised care planning [17].

However, despite these evidence based guidelines screening is not always routinely carried out or monitored [14, 18]. Subsequently, there is a large body of evidence reporting unmet needs in those affected by cancer relating to physical, psychological, practical and/or social factors [19–21]. What is more, it has been suggested that the majority of unmet needs are beyond the function of services that are primarily designed to focus on the medical aspects of care [22]. It is against this backdrop that a community based cancer service in Glasgow called ‘Improving the Cancer Journey’ (ICJ) was developed.

‘Improving the Cancer Journey’ (ICJ) is led by by Glasgow City Council (the legislative body that governs the city) and the main partner in funding and support is a UK charity called Macmillan Cancer Support. Other partners include the National Health Service (NHS) and a range of organisations operating in health and social care. The service sends individuals who are newly diagnosed or have had a disease reoccurrence a letter of invitation. This letter invites them to have a Holistic Needs Assessment (HNA) with a named link officer. This is offered in a community setting, such as a local library or the individuals home rather than a clinical setting where a HNA is traditionally carried out. However, individuals can also be referred into the service by their health professional or they can self-refer.

Holistic needs assessment is a common method of screening for distress in cancer care [23]. It is defined as follows:

*“Holistic needs assessment is a process of gathering and discussing information with the patient and/or carer/supporter in order to develop an understanding of what the person living with and beyond cancer knows, understands and needs. This holistic assessment is focused on the whole person, their entire well-being is discussed – physical, emotional, spiritual, mental, social, and environmental. The process culminates when the assessment results are used to inform a care plan.”* [24]

**Fig. 1:**
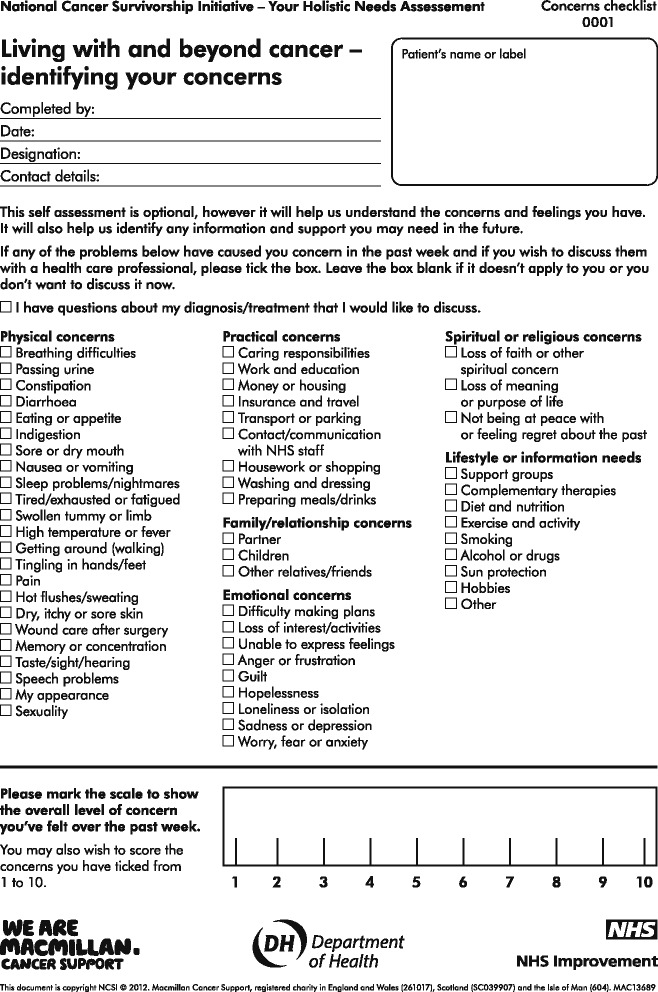
Holistic Needs Assessment- The Concerns Checklist

The assessment allows the link officer to understand what the central concerns are for that individual (Fig. 1). The individual completing the assessment is asked to consider on a scale of 1–10 how much the issue identified is causing them concern. This allows the service to identify if an individual’s level of concern changes over time. The link officer is a member of staff employed by Glasgow City Council with a background in either housing and/or social care. The service also has a seconded Cancer Nurse Specialist who provides clinical support to the team, a patient reference group and a clinical psychologist who provides supervision for the link officers.

Through administering a HNA the link officer can devise an individualised care plan and provide guidance and support to the person affected by cancer in order to access local services in relation to their needs. The link officer revisits each case approximately every 4 to 6 weeks, (or sooner depending on need) closing the case when they agree with their client that they require no further assistance from the service. The service also offers support to carers. For the purposes of this research a ‘carer’ refers to anyone who cares unpaid for the person with a cancer diagnosis. For example, this may be their spouse, friend or next of kin.

Improving the Cancer Journey (ICJ) was launched in February 2014 with a pilot phase focusing on five cancer groups. This service was then extended to all cancer types. It has been commissioned for 5 years. As of July 2016 the service has supported 1801 people. 52 % are female and 48 % are male. It supports most people in the 50–64 age bracket and 93 % of those who have accessed the service identify as having a white ethnicity. The most common primary diagnosis is lung cancer, followed by breast cancer.

This research was commissioned to focus on *impact* and *process* issues relevant to a) the individuals in receipt of the service, b) the service providers, and c) the wider culture. This research runs alongside the life of the programme for the next 5 years and is designed to draw out learning from the programme in an ongoing way.

### Aim and objectives

The overarching aim of this study is to understand if and how ICJ impacts upon the experiences of people living with and affected by cancer. There are 2 objectives:

1. To identify the impact of ICJ on those diagnosed with cancer
2. To understand the process of ICJ as experienced by
  ○ Patients and carers in receipt of the service
  ○ The service providers, and
  ○ The wider culture.

Hypotheses are:

1. Individuals who have engaged with ICJ will report significantly different mean scores on patient activation measures than those who have not.
2. Individuals who have engaged with ICJ will report significantly different mean scores on quality of life measures than those who have not.
3. Individuals who have engaged with ICJ will report significantly different mean scores on social support than those who have not.
4. Self -reported experience of ICJ will be significantly associated with scores on quality of life measures, patient activation scores and support.

## Design/Methods

This study uses a sequential mixed methods design. The main rationale for using mixed method research is triangulation of the data.

### Data

This study will incorporate the following data gathering methods across the 5 years:

#### Quantitative data

- Validated questionnaires (PAM-13, FACT-G, EQ5D-3L, MOS-SSS), analysis of routinely collected service data and self-reported experience of ICJ. See ‘Measures’ below for detail.

#### Qualitative data

- Free text
- Interviews with patients and carers
- Reflective diaries for the link officers
- Observation of the link officers.

### Intervention

Holistic needs assessment and targeted support. A link officer conducts a holistic needs assessment (HNA) with a person affected by cancer. A care plan is then co-constructed between the worker and the person. Actions from the care plan may include referral to an external agency, referral back to the health service, signposting to another service or providing self-management information. The care plan is revisited at each meeting between the link officer and the person affected by cancer until issues are resolved.

### Eligibility criteria

#### Patients and carers who have used ICJ

For inclusion in this research the patient and/or carer will have received the intervention.

Inclusion criteria:

- Over 24 years old (this is a requirement of the ICJ service)
- Live in Glasgow
- Diagnosed with cancer or supporting someone with a cancer diagnosis
- Had a HNA with a link worker as part of the ICJ service

Exclusion criteria:

- Professional carer (eg paid to provide care)
- Person deemed incapable of consenting to participate as defined by the Adults with Incapacity Act (2000)
- Any reason which in the opinion of the clinician/investigator interferes with the ability of the patient to participate in the study

#### Patients who have been offered but not received ICJ

This study will also include data from a sample of individuals diagnosed with cancer that have been offered ICJ but not taken up the offer (for any reason). The reason for including this non ICJ sample is to a) understand why people have not taken up the service and b) to compare activation, quality of life, health status and support levels according to whether people had received ICJ or not.

#### Link officers

There are currently 7 link officer employed full-time by this service. All will be included in the study unless they choose not to participate.

#### Other stakeholders

There is a range of partners involved in this service as described in the background section. In addition, there are various agencies and services that the patients and carers may engage with as a function of this service, such as psychological services and third sector organisations. These stakeholders will be invited to participate in annual workshops.

### Measures

Participants who have used ICJ and the comparable sample of participants who have been offered but not received ICJ will receive a postal questionnaire. The questionnaire contains validated measures of patient activation, quality of life, health status, social support, a free text box and space to prove contact details if they wish to take part in qualitative follow-up interviews.

#### Patient activation

Patient activation is a behavioural concept relating to an individual’s self-management needs, abilities and priorities. The patient activation measure short form (PAM-13) is a 13-item Likert measure constructed to identify different levels of patient activation. These levels have been used to estimate costs in relation to service use such as hospital admissions and accident and emergency usage [25]. A related benefit of this measure is that the levels are a useful indicator of the types of support individuals may require form health professionals to engage in self-management [26].

#### Quality of life

Participants will complete the functional assessment of cancer therapy-general (FACT- G) and the EQ-5D [27]. FACT-G is a validated measure of quality of life in a general cancer population [28, 29]. EQ-5D is a standardised instrument for measuring economic preferences for health states. It is in widespread use in many countries and provides a simple descriptive profile and index value for health status [30].

#### Social support

The Medical Outcomes Study Social Support Survey (MOS SSS) is a validated measure of perceived social support that was developed for patients with chronic conditions [31]. It encompasses several domains of support including tangible support, emotional support and positive support.

#### Open-ended question

At the end of the questionnaire a separate free-text box asks ‘If you have anything else you would like to tell us about living with cancer please do so here’. The aim is to analyse the content for any themes relating to individuals cancer experiences.

#### Self-reported experience of ICJ

The service sends every individual an evaluation questionnaire following their final visit. It asks a mixture of closed and open-ended questions relating to their motives for taking up the service, their experiences with the link officer and what impact the service may have had in terms of reducing concerns and fostering self-management. Where available this data will be matched up with the data from our questionnaire to examine if there are any associations between self-reported experience of ICJ and patient activation, quality of life and social support.

### Recruitment

#### Individuals who have used ICJ

At the initial meeting where the HNA takes place individuals are asked for their consent to share their data. For those who have consented ICJ keep a record of contact details for all their clients. All individuals who meet the inclusion criteria will be invited to participate. Participants will receive a study pack through the post. This will contain a welcome letter, participant information sheet, consent form and questionnaire booklet. If they wish to take part they will be provided with a stamped addressed envelope to return the questionnaire and consent form.

A final question on the questionnaire asks if they wish to take part in follow up interviews with a researcher. If they do, they will be asked to provide their contact details so the researcher can arrange this. The research team will recruit patients and carers this way. In year one the target is to interview 20 patients and carers who have used ICJ and indicated on the questionnaire that they wish to take part. We will purposively seek to recruit consenting participants who have experienced a wide range of outcomes as evidenced in their questionnaire responses. We will target different cancers and cancer stages to help obtain a broad sample. Written informed consent will be obtained for all interview participants. The process will be repeated at 2.5 and 4 years.

#### Individuals who have been offered but not received ICJ

A stratified sample based on gender, age, cancer type and socio-demographic band will be developed based on the ‘individuals who have used ICJ’ sample. The service will also post the study pack on behalf of the research team to this sample alongside a reminder that ICJ is still available to them should they so wish.

#### Link officers

The research team will provide an information session for the 7 link officers to detail the study aims and what their contribution will be. This will be supplemented with a participant information sheet. This process will be managed sensitively. As the link officers are closely aligned to this service they may find it difficult to say no to participation. Therefore, we will encourage them to take time over their decision and discuss it will their line manager. As with all participants we will remind them that they may withdraw at any point and this will not affect their working environment in any way. Written informed consent will be obtained for all those who wish to take part.

### Analysis

*Objective 1 - Identify the impact of ICJ on those diagnosed with cancer*

Hypotheses are:

1. Individuals who have engaged with ICJ will report significantly different mean scores on patient activation measures than those who have not.
2. Individuals who have engaged with ICJ will report significantly different mean scores on quality of life measures than those who have not.
3. Individuals who have engaged with ICJ will report significantly different mean scores on social support than those who have not.
4. Self -reported experience of ICJ will be significantly associated with scores on quality of life measures, patient activation scores and support.

#### Power analysis

There is no evidence to support effect size estimates so a moderate effect size of ICJ (d = 0.5) was adopted. Alpha was set to 0.0167 to allow for testing three different hypothesised mean differences (Hypotheses 1–3) on the same samples. With power set at 95 % this returned an estimated required total sample size of 264 (132 in each group) using G*Power version 2.

For the fourth hypothesis (self-reported experience of ICJ will be associated with scores on quality of life measures, patient activation scores and support) we ran an exact test on G*Power (version 2) running a correlation: bivariate model with the following assumptions: one tailed, hypothesised correlation 0.3, alpha error .05, power 95 %. This returned a sample size of 115. Whilst we hope to obtain a larger sample in order to conduct subgroup analyses, the required sample appears achievable.

The data will first be tested for outliers using boxplots and for normality using QQ plots. If normality is found, homogeneity of variance between groups will be tested with Levene’s test. Subsequent calculations will be based on the outcomes of these assumption tests. If normality and homogeneity of variance are established mean scores (from the PAM-13, FACT-G, EQ5-D and the MOS Social Support Survey) will be compared between the ICJ and non-ICJ group using *t*-test. If normality and/or homogeneity of variance cannot be established, a corresponding nonparametric method will be employed.

*Objective 2: To understand the process of ICJ as experienced by:*

- ○ Patients and carers in receipt of the service
- ○ The service providers, and
- ○ The wider culture

#### Semi-structured interviews

Interview schedules have been designed to align with the content of questionnaire. Specifically, they will provide richer insight into the relationship between the use of ICJ, wider service utility, satisfaction with support, quality of life and self-management/patient activation. Interviews will be conducted at 1 year, 2.5 years and 4 years. We plan to use a new sample in each case, targeted to best provide context to the data from the most recent questionnaire. However, we also plan to maintain contact and re-interview consenting patients and carers interviewed previously in order to obtain longitudinal data on their experiences. The intention is to record peoples’ experiences in depth as they move through their cancer experience (which may for example include a return to the ICJ service). This will develop understanding around how the needs of people living with cancer may change and evolve over different stages and what implication this may have for the service.

Analysis of the interviews will be conducted using framework analysis [32]. The approach involves a systematic process of categorising data according to identified issues and themes, providing a rich map of the observations as a whole [33, 34].

The five steps in this analysis are distinct but interrelated. They are:

1. Familiarisation
2. Conceptualisation of themes
3. Application of themes to the data
4. Rearranging the data according to themes
5. Mapping which enables the data to be interpreted as a whole

Two researchers will read all the transcripts and generate initial codes. These will then be formed into themes. Member checking will then take place. This involves sending the participants a copy of the preliminary analysis and asking for their opinion. Any suggestions can then be integrated into stage four. A discussion between the two authors will then take place until the two researchers feel they have reached consensus. The transcripts will then be re-read to ensure that the themes remain relevant. This analysis will be written into an annual report and shared at yearly patient/carer and stakeholder workshops.

#### Observations

We will observe the link officer team to examine the service perspective. A structured observation method will be used. The purpose of the observation is to record the physical and verbal behaviour of the link officers and their clients in order to see how each visit is conducted in terms of how the assessment is introduced, delivered and turned into action. An observation schedule was derived from existing literature and the aims and theory of this study to ensure that observations are systematically recorded and analysed [35]. Each link officer will be observed on a rotational basis for one, 3-h observation period every Wednesday morning. The total observation period will last for 3 months a year, totalling a minimum of 2 observations per participating link officer per year. Analysis of the field notes will also be conducted using framework analysis.

#### Reflective diaries

Each link officer will be issued with a reflective diary to examine the service perspective. The diary will contain prompt questions relating to: what went well today, what didn’t go well, learning gains and needs. Applying the same structure to every diary will provide insight into any shared or differing experiences. A fixed assessment schedule will be used, meaning the link officer will be asked to report on events and experiences at the end of every day during the working week. The diary transcripts will be analysed using content analysis in order to identify common and/or unusual themes. In summary, we wish to ascertain what a link officers ‘typical’ experience is like, how do these experiences differ, what processes (if any) underlie these differences and how these experiences may change over time. This is the most useful method of diary analysis in this type of study [36].

Field notes from the interviews, observations and diary entries will be synthesised in order to combine rich data on the role of the link officer, the experience of the patient and the process elements of ICJ. All data will be analysed within NVivo version 10 for consistency. Main themes emerging from the data will be discussed with participants on an ongoing basis in order to verify preliminary findings [37].

## Discussion

This study examines how a community-based service, delivering a holistic method of support may impact on individuals affected by cancer. This innovative service is a UK first. Traditionally holistic needs assessment is administered by a health professional, such as a cancer nurse specialist or oncologist in an outpatient clinic setting. This service moves the assessment into the community and is delivered by professionals with a housing and social care background. This shift allows the link officers to focus on concerns that are not appropriately addressed by the health service such as housing and financial issues while referring any clinical issues back to the oncology team. This supports evidence highlighting that frequently reported unmet needs of people with cancer relate to practical issues [38]. Further, the aim is to target any concerns as soon as possible as unresolved concerns at an earlier stage in the cancer experience can have an impact on later psychological adjustment [39]. Therefore, addressing all areas of need at an early stage seems to be the most ethical form of care.

This mixed methods study will explore impact and process issues from the perspective of both those who deliver and receive the service. Using triangulation allows examination of this service from multiple dimensions to explore convergence and complementarity in order to increase the credibility of the findings. This will provide crucial evidence in terms of the future sustainability of the programme and potentially provide a model of care for other regions to follow. Quantitative data will provide an insight into the impact of a supportive service on patient activation, quality of life, health status and levels of perceived support, outcomes that are both fundamental and meaningful for service providers, funders and people with a cancer diagnosis. Qualitative data will add depth to these findings through exploring how and why this service may, or may not contribute to these outcomes. This will provide a unique insight into the relationship between psychological, social and physical issues for those affected by cancer.

This protocol has limitations. We acknowledge Glasgow’s distinctive demography and the impact this may have on the generalizability of the findings. This is why we have proposed to evaluate the service from three levels; patient/carer, link officer and wider stakeholders to examine both individual and cultural factors to determine what factors are unique to the local area, what elements may be transferable and how this corresponds to wider findings relating to how and why this service may improve the lives of those affected by cancer. For comparative purposes we will distribute the questionnaire to a matched sample of individuals who also live in Glasgow, have been offered the ICJ service but decided not to take it up. As this sample live in Glasgow it minimizes any possible confounding variables from, for example, recruiting individuals who are cared for under a different health board. We hypothesise that those who have used the service with have significantly better outcomes than those who have not. However, we do not know why this group of individuals decided not to take up the service. It is possible that they are already well supported meaning there will be no difference in their outcomes. We will explore their reasons for not taking up the service along with descriptive statistics to provide further context to these findings.

Health policy in many countries has prioritised detecting and screening for psychological needs relating to cancer. Yet, despite this, distress is very common in those affected by cancer, across diagnoses and the disease trajectory. This service takes a proactive approach to screening drawing on a psychosocial holistic model of care to offer everyone in the city of Glasgow a personalised assessment right from diagnosis until whatever point that individual decides they no longer require the support. It is this element of care that is unique. The personalised approach provided by the link officers is at the centre of this service. Consequently, a key part of this research will focus on their experiences delivering the service. This will gather evidence that highlights how their skills and interactions with their clients shape the service but also how the service managers can support the link officers. This may be through the identification of training needs or recognition of the need to incorporate regular supervision into their working pattern to cope with the demands of working in an emotionally charged environment.

If the outcomes are favourable there is potential for broader dissemination. Currently, there are early plans to replicate this model within Scotland and there has been interest from several regions in England. This is the first independent evaluation of what impact this service and subsequently others that follow it, may have on the lives of those affected by cancer. Therefore, the findings from this study are likely to be relevant to policy makers, service providers, people affected by cancer and could influence the provision of holistic care both nationally and internationally.

## Abbreviations

EQ-5D: EuroQol 5 Dimension
FACT-G: Functional assessment of cancer therapy- general
HNA: Holistic needs assessment
ICJ: Improving the cancer journey
MOS-SSS: The medical outcomes study social support survey
PAM-13: Patient activation measure

## References

[1] WHO. Cancer Fact Sheet No. 297 [Internet]. 2015 [cited 2016 Feb 10]. Available from: http://www.who.int/mediacentre/factsheets/fs297/en/. Accessed 9 March 2016.

[2] Cancer Research UK [Internet]. 2016 [cited 2016 Feb 10]. Available from: http://www.cancerresearchuk.org/health-professional/worldwide-cancer-statistics. Accessed 9 March 2016.

[3] Torre LA, Bray F, Siegel RL, Ferlay J, Lortet-tieulent J, Jemal A (2015). Global Cancer Statistics, 2012. CA a cancer J Clin.

[4] Scottish Executive (2001). Cancer in Scotland: Action for change.

[5] Gray L, Leyland AH (2009). Is the “Glasgow effect” of cigarette smoking explained by socio-economic status? A multi-level analysis. MBC Public Heal.

[6] The Scottish Government. Cancer [Internet]. 2016 [cited 2016 Mar 17]. Available from: http://www.gov.scot/Topics/Health/Services/Cancer. Accessed 9 March 2016.

[7] Information Services Division (2015). Cancer in Scotland.

[8] The Scottish Government (2012). Scottish Index of Multiple Deprivation 2012.

[9] Landy R, et al. Do socio-economic, behavioural and biological risk factors explain the poor health profile of the UK's sickest city?. Journal of Public Health. 2012;34(4):591-598. 10.1093/pubmed/fds020 22421460

[10] Zabora J, BrintzenhofeSzoc K, Curbow B, Hooker C, Piantadosi S (2001). The prevalence of psychological distress by cancer site. Psycho-Oncology..

[11] Gaugler JE, Hanna N, Linder J, Given CW, Tolbert V, Kataria R (2005). Cancer caregiving and subjective stress: A multi-site, multi-dimensional analysis. Psycho-Oncology..

[12] Departement of Health (2012). Improving outcomes: a strategy for cancer.

[13] National Institute for Clinical Excellence. Guidance on Cancer Services. Improving supportive and palliative care for adults with cancer. The Manual. 2004.

[14] NCCN (2003). Distress management. Clinical practice guidelines.

[15] NHS Improvement. Stratified pathways of care. From concept to innovation [Internet]. 2012 [cited 2016 Mar 17]. Available from: http://www.ncsi.org.uk/what-we-are-doing/risk-stratified-pathways-of-care/. Accessed 9 March 2016.

[16] Stanciu M, Morris C, Mankin M. A pilot randomised controlled trial personalised care after treatment for prostate cancer (TOPCAT-P): nurse led holistic-needs assessment and individualised psychoeducational intervention: study protocol. BMJ Open. 2015. 10.1136/bmjopen-2015-008470 PMC4486944 26112224

[17] Departement of Health. Personalised care planning an “at a glance” guide for healthcare professionals. 2011. https://www.gov.uk/government/uploads/system/uploads/attachment_data/file/215946/dh_124048.pdf. Accessed 9 March 2016.

[18] Mccarter K, Britton B, Baker A, Halpin S, Beck A, Carter G, et al. Interventions to improve screening and appropriate referral of patients with cancer for distress: systematic review protocol. BMJ Open. 2015. 10.1136/bmjopen-2015-008277 PMC4577928 26391631

[19] Hagedoorn M, Sanderman R, Bolks HN, Tuinstra J, Coyne JC (2008). Distress in couples coping with cancer: a meta-analysis and critical review of role and gender effects. Psychol Bull.

[20] Sanders SL, Bantum EO, JEO Ã, Thornton AA, Stanton AL (2010). Supportive care needs in patients with lung cancer. Psycho-Oncology.

[21] Wells M, Cunningham M, Lang H, Swartzman S, Philp J, Taylor L (2015). Distress, concerns and unmet needs in survivors of head and neck cancer: a cross-sectional survey. Eur J Cancer Care..

[22] Soothill K, Morris S, Harman J, Francis B, Thomas C, McIllmurray MB (2001). The significant unmet needs of cancer patients: probing psychosocial concerns. Support Care Cancer.

[23] Young J, Cund A, Renshaw M, Quigley A (2015). Improving the care of cancer patients : holistic needs assessment. Br J Nurs.

[24] National Cancer Survivorship Initiative (2013). Assessment & Care Planning Definitions.

[25] Kinney RL, Lemon SC, Person SD, Pagoto SL, Saczynski JS (2015). The association between patient activation and medication adherence, hospitalization, and emergency room utilization in patients with chronic illnesses: A systematic review. Patient Educ Couns.

[26] Kidd L, Lawrence M, Booth J, Rowat A, Russell S. Development and evaluation of a nurse-led, tailored stroke self-management intervention. BMC Health Serv. Res. BMC Health Services Research; 2015;1–12. 10.1186/s12913-015-1021-y PMC4559282 26335777

[27] EuroQol [Internet]. [cited 2016 Mar 18]. Available from: http://www.euroqol.org/about-eq-5d.html. Accessed 9 March 2016.

[28] Webster K, Cella D, Yost K (2003). The Functional Assessment of Chronic Illness Therapy (FACIT) Measurement System: properties, applications, and interpretation. Health Qual Life Outcomes.

[29] Fairclough DCD (1996). Functional Assessment of Cancer Therapy (FACT-G): Non-response to individual questions. Qual Life Res.

[30] Pickard AS, Wilke CT, Lin HW, Lloyd A (2007). Health utilities using the EQ-5D in studies of cancer. Pharmacoeconomics.

[31] Sherbourne CD, Stewart A (1991). The MOS social support survey. Soc Sci Med.

[32] Ritchie J, Spencer L. Qualitative data analysis for applied policy research. The qualitative researcher’s companion. 2002;573:305-29.

[33] Ward DJ, Furber C, Tierney S. Swallow V. Using Framework Analysis in nursing research: a worked example. J Adv Nurs. 2013;69(11):2423–31. 10.1111/jan.12127 23517523

[34] Srivastava A, Thomson SB (2009). Framework Analysis: A qualitative methodology for applied policy research. J Adm Gov.

[35] Mulhall A (2003). In the field: Notes on observation in qualitative research. J Adv Nurs.

[36] Bolger N, Davis A, Rafaeli E (2003). Diary methods: capturing life as it is lived. Annu Rev Psychol.

[37] Walker D, Myrick F (2006). Grounded theory: an exploration of process and procedure. Qual Health Res.

[38] Harrison JD, Young JM, Price MA, Butow PN, Solomon MJ (2009). What are the unmet supportive care needs of people with cancer ? A systematic review. Support Care Cancer.

[39] Petty L, Lester J (2014). Distress Screening in Chronic Disease: Essential for Cancer Survivors. J Adv Pract Oncol.

